# Wood Chemical Composition in Species of Cactaceae: The Relationship between Lignification and Stem Morphology

**DOI:** 10.1371/journal.pone.0123919

**Published:** 2015-04-16

**Authors:** Jorge Reyes-Rivera, Gonzalo Canché-Escamilla, Marcos Soto-Hernández, Teresa Terrazas

**Affiliations:** 1 Departamento de Botánica, Instituto de Biología, Universidad Nacional Autónoma de México, Mexico City, Mexico; 2 Unidad de Materiales, Centro de Investigación Científica de Yucatán, Mérida, Yucatán, Mexico; 3 Programa de Botánica, Colegio de Postgraduados en Ciencias Agrícolas, Montecillo, Estado de México, Mexico; Purdue University, UNITED STATES

## Abstract

In Cactaceae, wood anatomy is related to stem morphology in terms of the conferred support. In species of cacti with dimorphic wood, a unique process occurs in which the cambium stops producing wide-band tracheids (WBTs) and produces fibers; this is associated with the aging of individuals and increases in size. Stem support and lignification have only been studied in fibrous tree-like species, and studies in species with WBTs or dimorphic wood are lacking. In this study, we approach this process with a chemical focus, emphasizing the role of wood lignification. We hypothesized that the degree of wood lignification in Cactaceae increases with height of the species and that its chemical composition varies with wood anatomy. To test this, we studied the chemical composition (cellulose, hemicellulose, and lignin content) in 13 species (2 WBTs wood, 3 dimorphic, and 8 fibrous) with contrasting growth forms. We also analyzed lignification in dimorphic and fibrous species to determine the chemical features of WBTs and fibers and their relationship with stem support. The lignin contents were characterized by Fourier transform infrared spectroscopy and high performance liquid chromatography. We found that 11 species have a higher percentage (>35%) of lignin in their wood than other angiosperms or gymnosperms. The lignin chemical composition in fibrous species is similar to that of other dicots, but it is markedly heterogeneous in non-fibrous species where WBTs are abundant. The lignification in WBTs is associated with the resistance to high water pressure within cells rather than the contribution to mechanical support. Dimorphic wood species are usually richer in syringyl lignin, and tree-like species with lignified rays have more guaiacyl lignin. The results suggest that wood anatomy and lignin distribution play an important role in the chemical composition of wood, and further research is needed at the cellular level.

## Introduction

**Fig 1:**
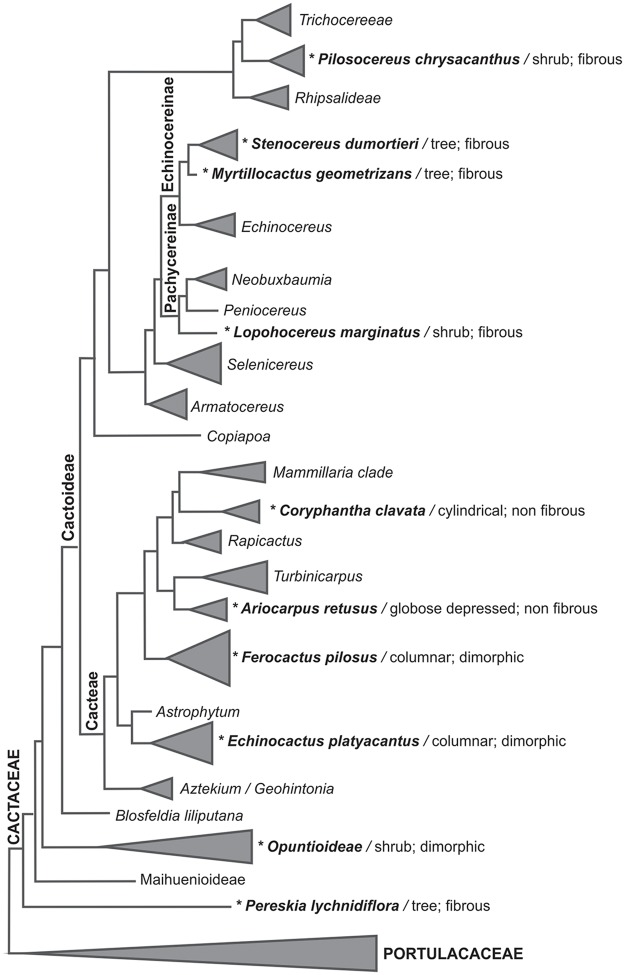
Phylogeny of Cactaceae. Asterisks indicate the analyzed species, which represent from the early derived taxa to the more derived ones, the different stem morphologies (tree, shrub, columnar, cylindrical, globose depressed) and the types of wood (fibrous, non fibrous, dimorphic). Topology based on and modified from references [58], [59].

Within angiosperms, the Cactaceae family is one of the most variable in terms of morphology and stem size, and this diversity is correlated with the anatomical features of the wood (Fig 1). Members of Cactaceae have both fibrous and non-fibrous wood [1] and are also dimorphic in the Cactoideae subfamily [2]. Cactaceae have tree-like species (hereafter called taller species) that are primarily fibrous, as observed in *Pereskia lychnidiflora* A.P. de Candolle, *Stenocereus dumortieri* (Scheidw.) Backeb., and *Opuntia streptacantha* Lemaire. The cylindrical, globose or globose-depressed species (hereafter called smaller species) have wood with abundant wide-band tracheids (WBTs) or with few fibers and parenchyma, such as *Coryphantha clavata* (Scheidw.) Backeb., as well as others with dimorphic wood (the change from WBTs to fibers as the predominant cell type) in medium-sized species such as *Echinocactus platyacanthus* Link & Otto and *Ferocactus histrix* (DC.) G.E. Linds. [3], [4].

The wood anatomy in Cactaceae is well documented, and several authors have noted a positive correlation between the characteristics of the wood (e.g., length and diameter of the fibers or vessel elements) and the plant morphology in terms of the support required to support the stem [4]-[8]. In some species of Cacteae, the traits of secondary xylem and the cell types present in the wood vary with the size of the species [9]. WBTs play a key role in the biology of cacti because these specialized cells occur in almost all species and may or may not persist through the life of the plant: in species with dimorphic wood, the vascular cambium stops producing WBTs and produces fibers. This is a unique process to cacti and is related to the aging of individuals and the improvement of stem support [10], [11]. It is important to note that these cells are structurally different from tracheids or fiber-tracheids of other linages because WBTs have thin primary walls and annular or helical secondary walls that project deeply into the tracheid lumen. It remains unknown if the chemical features of the wood in species with an abundance of WBTs or with dimorphic wood are similar to those of the fibrous species and if these are related to the stem morphology.

In recent years, studies have focused on the wood chemical composition of economically important species, and it is well documented that properties such as strength, rigidity and defense are principally enhanced by the lignin of the secondary cell walls and the anatomical features of the wood [12], [13]. In addition, previous studies have shown that the percentage and composition of lignin vary across different plant groups (i.e., 19–24% in angiosperms; 27–33% in gymnosperms) [14]-[17]. In dicots, lignin is usually composed of moieties of guaiacyl (G) and syringyl (S); in monocots, lignin also occurs as *p*-hydroxyphenyl (H) units. Lignin in gymnosperms and pteridophytes is richer in guaiacyl [13], [14], [18]. Similarly, the topochemical distribution of lignin in the wood varies with cell type. Previous research has shown that the fibers are richer in syringyl lignin (S), whereas guaiacyl lignin (G) is more concentrated in the secondary walls of vessels [19]-[21]. However, the variation in the lignification of the wood is influenced by anatomical characteristics. For example, the woody angiosperms have fibers, vessels and parenchyma and contain less lignin than gymnosperms whose wood contains only tracheids and scarce parenchyma [22], [23]. Because tracheids and fibers constitute the majority of wood cells in gymnosperms and angiosperms, respectively, they provide a significant contribution to the physical and chemical properties of the wood and stems [23]. However, the role of lignification in the support of the stem within Cactaceae is not currently well understood because most species develop WBTs that differ structurally from cells present in other lineages; to date, these WBTs have not been chemically analyzed. Here, we study the relationship between stem support and wood anatomy in Cactaceae with a chemical focus, emphasizing the role of wood lignification.

We hypothesized that the degree of wood lignification increases with the height of the species and that its chemical composition varies according to the anatomical characteristics of the wood in terms of the abundance of WBTs or fibers. To test this, we quantified the chemical components of wood in thirteen species of Cactaceae with different sizes and different growth forms. We characterized the lignin by Fourier transform infrared spectroscopy (FTIR) and high performance liquid chromatography (HPLC) and determined whether the percentage of lignin is related to the abundance of a specific cell type or stem morphology.

## Materials and Methods

We studied the chemical composition of wood in adult plants with contrasting growth forms belonging to thirteen species from three subfamilies of Cactaceae (Fig 1), representing from the early derived taxa to the more recent derived ones. According to the literature there is intraspecific variation in the chemical composition of wood [12]-[14], but it tends to be higher between taxa. We therefore evaluated interspecific variation with a reduce number of samples per species. Furthermore, a global analysis of wood details its chemical composition in species where the percentage of wood-forming cells is above 80% [23]. Within Cactaceae, wood anatomy in adults of the same species is very consistent [2], [3], [7], [12], [24]; therefore, the chemical composition is expected to be very similar.

In all species, the vascular cylinder was isolated, and the wood near the root (lower wood) was selected for chemical analyses because it has a greater accumulation of wood, as has been demonstrated for other cacti [4], [7], [8], [24]. For most taxa, we followed standard protocols to make transverse sections using the sliding microtome [7], [24]. For two species, *Ariocarpus retusus* and *Coryphantha clavata*, wood was embedded in paraffin according to the procedure of Loza-Cornejo and Terrazas [8]. All sections were double-stained with safranin-fast-green [8], to clearly differentiate the primary and secondary walls. In two dimorphic species (*E*. *platyacanthus* and *F*. *pilosus*), we examined the lignification in the radial and longitudinal directions to determine whether there was variation related to the different regions of the stem or the cell types within the wood. We separated the region near the pith and the region near the vascular cambium from the lower wood and analyzed them separately; we also analyzed the wood near the apex of the stem (upper wood). Because the wood is accumulated over the life of individuals to form the vascular cylinder, both the juvenile and adult stages of each individual are chronologically represented in the lower wood: the juvenile and mature wood is found closer to the pith and near the vascular cambium, respectively, *sensu* Panshin & De Zeeuw [25]. Similarly, the upper wood can be considered as a juvenile progression of maturity states [26]. Therefore, the lignification in the juvenile lower wood and mature lower wood represents the transition from WBTs to fibers in species with dimorphic wood, and the lignification of the upper and lower wood represents the juvenile and mature stages in fibrous species, respectively. Table 1 contains the morphological details and the wood regions sampled (i.e., upper, lower, juvenile and mature wood) for each species.

**Table 1:** Characteristics of individuals studied from thirteen species of Cactaceae, the regions of wood that were studied are indicated.

Individuals	Species	Height (m)	Wood Type	Stem	Region of wood	Category by size
2	*Ariocarpus retusus*	0.10 and 0.18	Non fibrous (WBT)	Depressed globose (T)	LW	Small
2	*Coryphantha clavata*	0.18 and 0.23	Non fibrous (WBT)	Cylindrical (T)	LW	Small
3	*Echynocactus platyacanthus*	0.26, 0.38 and 1.5	Dimorphic (transition*)	Columnar (R)	LWc,LWp and UW	Medium
1	*Ferocactus hamatacanthus*	0.7	Dimorphic	Cylindrical (TR)	LWc and UW	Medium
2	*Ferocactus pilosus*	0.36 and 0.63	Dimorphic (transition*)	Columnar (R)	LWc,LWp and UW	Medium
1	*Cylindropuntia imbricata*	3	Fibrous	Tree	LWc	Tall
1	*Lophocereus marginatus*	4.1	Fibrous	Tree (R)	LWc	Tall
2	*Myrtillocactus geometrizans*	2.7 and 4	Fibrous	Tree	LWc and UW	Tall
1	*Opuntia streptacantha*	5	Fibrous	Tree	LWc	Tall
2	*Pereskia lychnidiflora*	4	Fibrous	Tree	LWc, UW	Tall
1	*Pilosocereus alensis*	1.5	Fibrous	Columnar (R)	LWc	Medium
1	*Pilosocereus chrysacanthus*	5.9	Fibrous	Tree (R)	LWc	Tall
2	*Stenocereus dumortieri*	3.8 and 4.5	Fibrous	Tree (R)	LWc and UW	Tall

**Abbreviations.** Tuberculated (T), ribbed (R), tuberculated ribs (TR). Lower wood (LW): near to the vascular cambium (LWc), near to the pith (LWp). Upper wood (UW).

* In these dimorphic species occurs a change from WBTs wood in juvenile stages to fibrous wood in the mature ones.

The wood samples (the whole cylinder for *A*. *retusus* and *C*. *clavata* and only the region near the vascular cambium in the other species) were air-dried for one week and subsequently oven-dried at 50°C for 48 h. Wood chips from samples of each individual were obtained, oven-dried, and ground into sawdust using a Willey mill with a 40–60 mesh size. The sawdust samples (2 g for triplicate, see S1 Dataset for details and number of replicates) were subjected to sequential Soxhlet extraction with benzene-ethanol (2:1), ethanol (96%) and distilled water [27]. The extractive-free fraction was used for chemical composition analysis [28], [29].

### Klason lignin analysis/FTIR

The isolation of acid-insoluble lignin was performed according to the TAPPI Standard T 222 om-02 [28]. The extractive-free wood (0.25 g) was treated with a solution of 72% sulfuric acid (3.5 mL) at 18°C by 2 h, then diluted with distilled water (140 mL) and boiled for 4 h at a constant volume. Lignin was separated using a filtration funnel (fine) and oven-dried at 105 ± 3°C until reaching a constant weight. IR spectra of lignins were obtained from a Nicolet Protege 460 FTIR Spectrometer (Thermo Scientific, San Jose, CA, USA), following previously published protocols [27], [30], [31]. MID-IR spectra of lignin samples (5% (w/w) lignin in KBr) were recorded between 400 and 4000 cm^−1^ at a resolution of 4 cm^−1^ [27]. Fifty spectra were averaged per sample and analyzed using the OMNIC 5.1 software (Thermo Scientific).

### Determination of the S/G ratio by nitrobenzene oxidation/HPLC

The determination of the S/G ratio of each extractive-free wood sample was obtained by nitrobenzene oxidation according to Nunes et al. [29]. Approximately 0.2 g (oven-dried) of each wood sample together with NaOH aqueous solution (7 mL; 2 mol L^-1^) and nitrobenzene (0.5 mL) was loaded into a stainless steel reactor and heated to 170°C for 2.5 h; the procedure was repeated twice. The oxidized material was then extracted with chloroform (6 x 30 mL). After the first extraction, HCl was added (2.5 mL; 4 mol L^-1^) to the aqueous phase. The combined organic phases were concentrated with a Buchi R-114 evaporator at 40°C. The sample was transferred to a 50 mL volumetric flask and the final volume was reached using an acetonitrile/water solution (1:1 v/v). The solution was filtered in a regenerated cellulose membrane with a pore size of 0.45 μm. Next, this solution was analyzed by high performance liquid chromatography (HPLC) in an Agilent 1100 apparatus equipped with a variable wavelength detector G1314A with standard flow cell operating at a 280 nm wavelength, a G1310A isocratic pump and a G1328A manual injector with a Lichrosorb RP-18 (250 x 4.6 mm, 5 μm) reverse phase analytical column. The mobile phase was composed of acetonitrile/water (14:86 v/v), and the pH was adjusted to 2.6 with a trifluoroacetic acid (TFA) buffer. The column temperature was kept at 40°C, and a mobile phase flow of 1.5 mL min^-1^ was used. Vanillin and syringaldehyde standards (Aldrich, Milwaukee, WI, USA) were used for the quantification of the guaiacyl and syringyl unit derivatives, respectively. Calibration curves using vanillin and syringaldehyde standards were obtained in the concentrations of 0.375, 0.75, 1.125, and l.5 mmol L^-1^ for vanillin, and 0.825, 1.65, 2.475, and 3.3 mmol L^-1^ for syringaldehyde. The solutions were prepared in an acetonitrile/water mixture (1:1 v/v) at a pH of 2.6. The S/G ratio was calculated considering the yield suggested by Ohra-ahoa et al. [32], assuming that approximately 30% of the condensed guaiacyl units are converted to vanillin and that 90% of the syringyl units are converted to syringaldehyde due to its low proportion of condensation.

### Content of cell wall carbohydrates

Cellulose and hemicellulose were quantified following the method of Donnelly [33]. Glacial acetic acid (0.05 mL) and sodium chlorite (0.15 g) were added to a mixture of extractive-free milled wood (1 g) and water (14 mL) at 74°C with constant stirring. Carbon dioxide was bubbled through the mixture, and the addition of acetic acid and sodium chlorite was repeated every 15 min for a total of four additions. After 1 h, the mixture was cooled to room temperature, filtered through a medium pore filter, and washed with water until free of acid; lastly, the holocellulose was washed with acetone.

### Isolation of hemicellulose A

Holocellulose (0.60 g) was extracted with 10% potassium hydroxide (10 mL) by stirring overnight in an atmosphere of nitrogen; the extract was then filtered on a filter cloth and washed with water. The clear filtrate was adjusted to a pH of 5.0 with a 50% acetic acid solution and refrigerated overnight. Hemicellulose A was collected by centrifugation, and the supernatant liquid was removed and saved for isolation of hemicellulose B. The hemicellulose A was washed with water three times, suspended in water and freeze-dried. The alkali solubilization and reprecipitation of hemicellulose A was repeated twice. The final product was a white amorphous powder.

### Isolation of hemicellulose B

The supernatant liquid obtained in the separation of the HA was poured into three times its volume of 95% ethanol under stirring. The precipitate was collected as described for the hemicellulose A, except that the product was washed with 95% ethanol. The lyophilized product was reprecipitated twice by redissolving in alkali, adjusting the pH to 5.0 with 50% acetic acid, and precipitating with 95% ethanol.

The total hemicellulose (TH) was calculated by the sum of both yields (TH = HA + HB). The yield of cellulose (Ce) was obtained by subtracting the total hemicellulose from the holocellulose obtained initially, expressed as (Ho) (Ce = Ho—TH).

The wood chemical composition is shown in total percentage (% w/w). After each chemical analysis, the chemical composition of three replicates per sample of the lower wood was averaged; the standard deviation was within 10% of the mean in all cases (Table 2; S1 Dataset). Spearman’s correlation analysis was performed to detect associations between the different variables: lignin content, plant size and S/G ratio.

**Table 2:** Chemical composition of lower wood in mature individuals of thirteen species of Cactaceae.

Species	Category by size	Wood type	Extractives	Lignin				Cellulose	Hemicelluloses
				%	Syringyl	Guaiacyl	Ratio S/G	%	%
*Ariocarpus retusus*	Small	WBT	39.4	13.7	52	48	1.1	31	15.9
*Coryphantha clavata*	Small	WBT	22.7	14.2	80	20	3.9	41.7	21.4
*Echynocactus platyacanthus* *	Medium	Dimorphic	15.7	51.8	40	60	0.7	22	10.5
*Ferocactus hamatacanthus*	Medium	Dimorphic	28.6	54.4	92	8	11.7	12.2	4.8
Ferocactus pilosus*	Medium	Dimorphic	24.2	49.8	78	22	3.5	17.4	8.6
*Cylindropuntia imbricata*	Tall	Fibrous	12.4	51.3	30	70	0.4	23.8	12.6
*Lophocereus marginatus*	Tall	Fibrous	11.1	49.9	51	49	1.1	25.5	13.5
*Myrtillocactus geometrizans*	Tall	Fibrous	11.6	56.3	38	62	0.6	21	11.1
*Opuntia streptacantha*	Tall	Fibrous	28	45.1	53	47	1.1	17.6	9.3
*Pereskia lychnidiflora*	Tall	Fibrous	11.4	36	48	52	0.9	34.4	18.2
*Pilosocereus alensis*	Tall	Fibrous	20.6	53.1	57	43	1.3	17.2	9.1
*Pilosocereus chrysacanthus*	Tall	Fibrous	7.7	56.8	30	70	0.4	23.2	12.3
*Stenocereus dumortieri*	Tall	Fibrous	24.6	39.4	25	75	0.3	23.6	12.5

The contents are reported in the total dry weight percent (% w/w). The standard deviation in each case was less than 10% on average.

(S1 Table, S3 Dataset. Supporting information for the S/G ratios)

* Species where the content of lignin in the lower wood was different between adult individuals, the difference in the lignin content in adults of F. pilosus was ≈ 19% and in E. platyacanthus was ≈ 23%, the other components of the wood vary proportionately. Moreover, in these dimorphic species occurs a change from WBTs wood in juvenile stages to fibrous wood in the mature ones.

## Results and Discussion

### Lignification variation between species of different sizes

As previously hypothesized, the lignin content was variable between species: it was similar within small species but was lower than the taller species (Table 2). Statistical analyses revealed an association between plant size and lignin content (r_s_ = 0.505, *P* < 0.005). Small species, with a predominance of WBTs, had low lignin content (13.7% in *A*. *retusus* and 14.2% in *C*. *clavata*); this result is lower than has been reported for other angiosperms reported previously, such as white birch (*Betula papyrifera*, 19%). Species with a predominance of fibers had a much higher percentage of lignin than has been reported from other angiosperms and gymnosperms [23]. For example, the lignin percentage was 36–56.8% in the fibrous and dimorphic species in Cactaceae, compared with 24% in red maple (*Acer rubrum*) or 33% in canadian hemlock (*Tsuga canadensis*). The extent lignification in wood of Cactaceae confers a high chemical and mechanical resistance despite the lesser accumulation of wood relative to other woody plants. This is interpreted as a strategy related to the succulence and robustness of the stems. The lignin confers resistance to chemical degradation by fungi [34], [35] and provides the structural rigidity needed to the stem upright. It also provides resistance to the cell wall that counters the negative pressures generated in the conductive elements during transpiration [18], [36].

The lignin content of smaller species was similar to that of the lower wood from juvenile individuals of medium species (i.e., 13.7% and 9.7% in the lower wood of one adult of *A*. *retusus* and one juvenile of *E*. *platyacanthus*, respectively). The adult wood of *A*. *retusus* and the juvenile wood of *E*. *platyacanthus* are non fibrous with vessels in a matrix of WBTs. In smaller cacti, completely lignified cell walls are not needed because the turgor pressure plays a key role in supporting their stem [1]-[3], [10], [37], [38]. Thus, the low content of lignin, concentrated in the helical or annular thickenings of WBTs, is enough to ensure the integrity of cells under high turgor pressures and to confer flexibility and strength to the wood due to the expansion-contraction processes during long periods of water stress.

### Variation in individuals of different sizes belonging to the same species

In *E*. *platyacanthus*, we found that the proportion of lignin in the lower wood increased with the size of the individuals (26 cm = 9.7%, 38 cm = 28.8%, and 150 cm = 51.8%). Part of this drastic change in lignin content is associated with changes of wood as individuals increase in size. In species with dimorphic wood, such as *E*. *platyacanthus* and *F*. *pilosus*, a change occurs in the dominant cell type during the juvenile and adult stages: in the first stage there is predominance of WBTs and in the last stage the fibers are dominant; this change has been associated with the increase in size accompanying plant aging [39]. The lower lignin content in smaller individuals compared with the largest individuals in the same species shows that the increase in lignification with the age/size of individuals is associated with the change from WBTs to fibers. This property is characteristic of some Cactaceae species with dimorphic wood [1], [2], [10], [39].

### Juvenile/mature wood and longitudinal variation

As previously mentioned, the transition from WBTs to fibers was studied in *E*. *platyacanthus* and *F*. *pilosus* because the change has been linked to development and morphology. We found a higher percentage of lignin in the mature lower wood compared with the juvenile lower wood (i.e., 28.8% and 49.8% in fibrous wood near the vascular cambium and 11.2% and 12.0% (from S1 Dataset), near the pith where WBTs are abundant). These results, in addition to the increase in lignification with the size/age of individuals, confirm that the change from WBTs to fibers occurs to enhance the stem support. The abundance of WBTs during the juvenile stages of dimorphic species, in addition to the turgor pressure, contributes to the loss of wood lignification.

Lower wood, which supports more weight, was more lignified than the upper wood in all cases. The difference between the values from the lower wood (Table 2) and those from the upper wood (S1 Dataset) were 3.6% in *P*. *lychnidiflora*, 11.2% in *M*. *geometrizans*, 17.9% in *S*. *dumortieri*, 36.4% in *E*. *platyacanthus*, 41.5% in *F*. *hamatacanthus*, and 44.1% in *F*. *pilosus*. Gibson [3] and Niklas et al. [6] have noted that the increase in the length or diameter of the fibers in the lower stems of species such as *M*. *geometrizans* and *P*. *pringlei* provides greater support for the heavy and succulent tissues of the branches. In the present study, we found that, in addition to the increase in cell size, the degree of lignification also increased in the lower wood. However, in taller fibrous species with non-succulent branches, such as *P*. *lichnidiflora*, the lignification of the lower wood was not necessarily as high as in other tree-like cacti with fibrous wood and succulent branches.

### Lignification and wood anatomy

**Fig 2:**
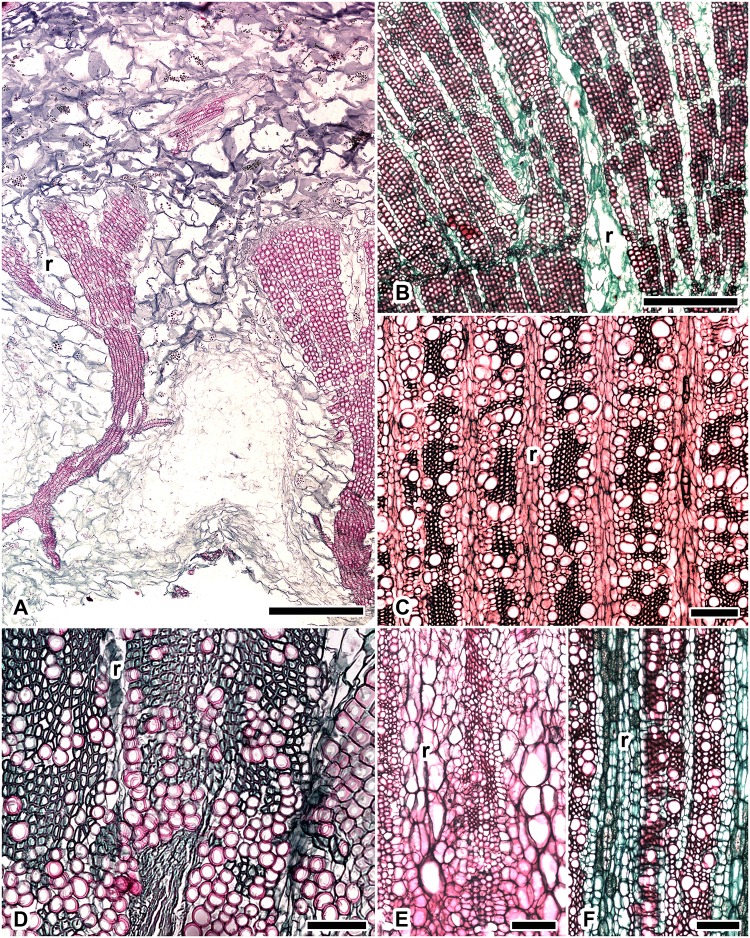
Non fibrous wood in species of Cacteae, cross sections near the vascular cambium. (A) *Ariocarpus retusus*: less lignified wood with vessels embedded in a matrix of WBTs separated by non lignified dilated rays; (B) *Coryphantha clavata*: wood with vessels embedded in a matrix of WBTs and narrow non lignified rays; (C) *Echinocactus platyacanthus*: wood with vessels embedded in a matrix of fibers and axial parenchyma with wider lignified rays; (D) *Ferocactus hamatacanthus*: wood with vessels embedded in a matrix of WBTs and fibers in similar proportions and a few narrow non lignified rays; (E) *Ferocactus pilosus*: wood with vessels embedded in a matrix of WBTs and lignified rays near the pith; (F) *Ferocactus pilosus*: wood with vessels embedded in a matrix of fibers and non lignified rays near the vascular cambium. Bar is 550 μm in A, B; 200 μm in C, E, F; 100 μm in D; r = ray.

**Fig 3:**
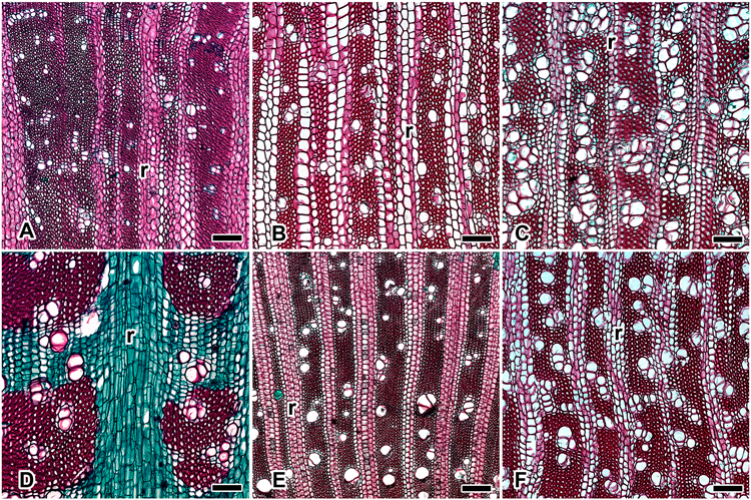
Fibrous wood in species of Cactaceae, cross sections, near the vascular cambium. Vessels embedded in a matrix of fibers with lignified rays except *Opuntia streptacantha*. (A) *Cylindropuntia imbricata*; (B) *Lophocereus marginatus*; (C) *Myrtillocactus geometrizans*; (D) *Opuntia streptacantha*: wood with vessels embedded in a matrix of fiber or parenchyma with intermixed WBTs and non lignified rays; (E) *Pereskia lychnidiflora*; (F) *Stenocereus dumortieri*. Bar is 200 μm; r = ray.

Most of the species with an abundance of WBTs (Fig 2) were less lignified than the fibrous species (Table 2). However, in *F*. *hamatacanthus*, the lignin content was similar to that of fibrous tree-like species with succulent stems (e.g., *M*. *geometrizans* and *P*. *chrysacanthus*) and greater than that in fibrous tree-like species with non-succulent stems such as *P*. *lychnidiflora* (Fig 3E). One explanation can be found in the wood anatomy: *F*. *hamatacanthus* has a cylindrical stem with tuberculate ribs, the epidermis is very soft and the wood has patches of fibers interspersed in a matrix of WBTs (Fig 2D). These features offer little support to the stem, suggesting that high lignification in *F*. *hamatacanthus* could be a strategy to largely absorb the body weight of the vascular cylinder. However, *P*. *lychnidiflora* has a tree-like growth form with very fibrous wood (Fig 3E), little cortical tissue, and non-succulent branches, i.e., the weight-bearing ability of the base of the stem is minor compared to that of other species with succulent branches. Therefore, the rigidity of the wood in non-succulent species is more favored by the volume of the support tissue (in terms of the amount of wood) than by the degree of lignification. This is in contrast to what occurs in species of cacti with succulent branches, in which the lignification, in addition to the amount of wood, confers stiffness to the stems [6].

In addition to the anatomical features of wood, stem morphological traits also appear to contribute to the support of the individual. For example, in species such as *E*. *platyacanthus*, the wood is mildly lignified due to the predominance of fibers, the numerous ribs, and the thickness of the epidermis; further, the extremely hard periderm confers stiffness and general support to the individuals similar to that of other cacti species with ribs [40]-[42].

### Wood anatomy and chemical composition of lignin relationship

#### Characterization of lignin by FTIR

**Fig 4:**
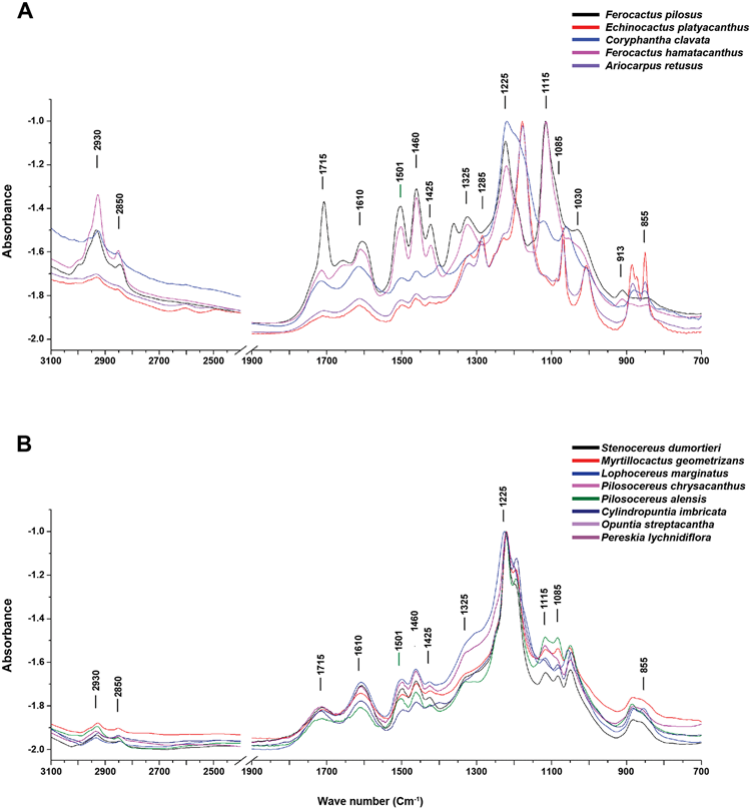
FTIR spectra of lignin in the lower wood of thirteen species of Cactaceae. (A) non fibrous species and dimorphic species belonging to the Cacteae tribe, showing varied lignin spectra; (B) fibrous species belonging from the early derived taxa to the more derived ones showing a highly conserved lignin spectra pattern. (S2 Dataset shows FTIR spectra matrix).

Bands previously assigned to guaiacyl (1030, 1285, 2930 cm^-1^) and syringyl (1225 and 1325 cm^-1^) lignins were observed in the FTIR spectra of Cactaceae (for references see Table 3). The bands in fibrous woods showed a uniform pattern; however, in dimorphic and non-fibrous woods the patterns were variable (Fig 4). The band at 1285 cm^-1^, assigned to the aromatic ring guaiacyl, was observed as a noticeable peak in two species (*A*. *retusus* and *E*. *platyacanthus*). Similarly, a shoulder at 1030 cm^-1^, assigned to in-plane deformation of C-H (guaiacyl), was observed in both *Ferocactus* species, whereas it was absent in the remaining species. The intensity of the peak found at 2930 cm^-1^, indicating the asymmetric vibration of CH_2_ (guaiacyl-syringyl), was higher in both *Ferocactus* species and *C*. *clavata*, whereas it was lower in the other species. The band at 1325 cm^-1^, assigned to syringyl lignin, was observed as a peak in Cacteae species except in *C*. *clavata*, in which it was observed as a shoulder similar to that in the fibrous species. The major peak at 1225 cm^-1^, assigned to the symmetric vibration of glucopiranosic ring (syringyl), was well defined in most species except for *E*. *platyacanthus* and *A*. *retusus*.

**Table 3:** Assignment of FTIR absorption bands in wood lignins.

Wave number (cm^-1^)	Assignments	References
2,930	CH2 asymmetric vibration (guaiacyl-syringyl)	[30], [31]
2,850	C-H stretching in methyl and methylene groups	[43]
1,715	Carbonyl stretching in conjugated ketone and conjugated carboxylic groups	[43], [44]
1,610	C = C aromatic ring vibration	[43]
1,501	C = C aromatic ring vibration (guaiacyl-syringyl)	[30], [31]
1,460	C-H asymmetric deformation	[44], [45]
1,425	Aromatic skeletal vibrations	[43]
1,325	Syringil ring breathing with C-O stretching	[45], [46]
1,285	C-O and glucopyranosic cycle guaiacylic symmetric vibration	[30]
1,225	C-O and glucopyranosic cycle syringilic symmetric vibration	[30]
1,115	C-O-C stretching and symmetric vibration of the ester linkage	[47]
1,085	C-O deformation in secondary alcohol and aliphatic ether	[43]
1,030	C-H in-plane deformation in guaiacyl and C-O deformation in primary alcohol	[48], [43]
913	**= CH** out-of-plane deformation in aromatic ring (guaiacylic-syringylic).	[31]
855	Aromatic C-H out-of-plane deformation	[43], [45]

The heterogeneity of lignin composition in Cacteae can be attributed to the variability of its anatomical features (Fig 2). These species may have dimorphic wood in which a transition occurs from WBTs to fibers or a unique combination of cell types. Changes within species can occur in different ways: a) an abrupt change in the early stages of development, such as in *E*. *platyacanthus*; b) a gradual change including more advanced stages, such as in *F*. *pilosus*; c) a persistent change resulting in a combination of both cell types throughout the life of individuals, such as in *F*. *hamatacanthus*; or d) the rare occurrence of fibers scattered in the matrix of WBTs or in the fascicle margins as in *C*. *clavata*. The last are difficult to see in cross sections and can only be clearly detected in macerated wood [9]. By contrast, the fibrous woods, in which the WBTs are absent (Fig 3), had a homogeneous composition similar to that reported in other typical woody dicots [21], [30], [31], [43], [46]. This indicates that a high abundance of fibers in the wood of Cactaceae induces a chemical composition similar to that from other woody lineages, independent of their phylogenetic position. Furthermore, the presence of WBTs in dimorphic wood modifies its global composition at different levels. Thus, it would be expected that other cacti with dimorphic woods and a predominance of WBTs would have a heterogeneous chemical composition, such as in members of Cacteae, and that species with highly fibrous woods would have patterns typical of other woody trees.

Variation in the lignin composition of species with dimorphic wood corresponds mainly to the bands associated with S (1225, 1325 cm^-1^) and G (1285 cm^-1^) lignins. In addition, there are very conspicuous bands that were not previously assigned in the analyses of lignins by FTIR, which appear to be related to fibrous (1194 cm^-1^) and dimorphic (1115, 1178 cm^-1^) woods. Specific chemical characterization studies are needed for these bands because they could be related to the composition of the secondary wall of the WBTs or be the result of an evolutionary process in the lignin biosynthetic pathway in Cactaceae, as has been suggested in studies of seed lignin in several species of this family [49].

#### S/G ratio by HPLC

We found that the syringyl/guaiacyl (S/G) ratio had an inverse association with species size (r_s_ = -0.640, *P* < 0.001) and was variable among the studied species (see Table 2). It ranged from 0.3 to 1.3 in ten of the thirteen species studied; particularly high values (3.5–11.7) were observed in three species in which the wood comprises a combination of fibers and WBTs as in *Ferocactus* and *Coryphantha*. Published data indicate that within angiosperms, the S/G ratio varies by species. For example, in the coihue (*Nothofagus dombeyi*), the ratio is 86:14, but the ratio in some monocots, such as *Lilium sitatissimum*, is 20:80 [14]. Notably, three of the studied species (*C*. *clavata*, *F*. *hamatacanthus*, *F*. *pilosus*) exhibited the highest ratio for syringyl higher than in most dicots, whereas one fibrous species, *S*. *dumortieri*, had the lowest values for syringyl, similar to some monocots and Pteridophytes [14]. The S/G variation in Cactaceae is interpreted as being related to the anatomy of the secondary xylem.

Previous studies of lignin in other lineages have correlated the S/G ratio with the proportion of condensed structures in lignin; the S units are linked by relatively labile ether bonds, whereas the G units are predominantly linked by more stable bonds, such as the C-C and biphenyl bonds, forming a highly condensed lignin [50], [51]. Thus, a low S/G ratio results in a degradation-resistant chemical structure due to the low proportion of S units [32], [52]. In addition, a number of authors have found that the S/G ratio appears to change from one morphological region to another: the secondary cell walls of fibers are richer in S lignin, whereas the secondary walls of vessels and the middle lamella are richer in G lignin [14], [15], [17], [23], [53]-[55]. However, in species in which tracheids or fibers constitute over 80% of the cells found in the wood, a global analysis reveals the composition of these cell types [23].

**Fig 5:**
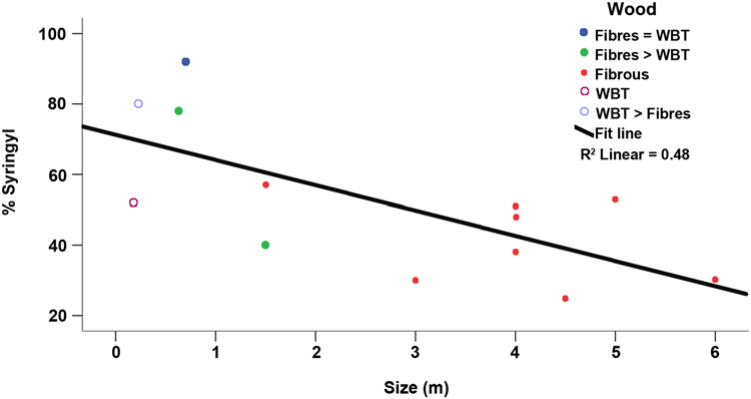
Relationship between plant species size and percentage of syringyl lignin. Each dot represents the species size given in S2 Table.

In Cacteae, the proportions of WBTs and fibers vary according to the species. In species of *Ferocactus* and *Coryphantha*, the presence of fibers and WBTs can persist into advanced stages of development; in the adult wood, one can find a combination of these cell types in different proportions. For example, in *F*. *pilosus*, rare WBTs can persist until later stages when the fibers are abundant; conversely, in *C*. *clavata*, the fibers are scarce and WBTs are abundant throughout the life of the individuals [9]. We observed similar S/G ratios in both *F*. *pilosus* and *C*. *clavata* (3.46 and 3.90, respectively). However, in *F*. *hamatacanthus*, the WBTs and fibers were interspersed in similar proportions (Fig 2D), which apparently led to a very high abundance of S lignin within the wood (Fig 5). The results indicate that the S/G ratio in these species is affected by the type of cell-cell interaction (i.e., the WBT-fiber junctions and their frequency throughout the wood). Thus, it is reasonable to observe similar S/G ratios in species in which the junction zones between WBTs and fibers are equally rare, such as in *C*. *clavata* and *F*. *pilosus*, and to observe high S/G ratios in species in which the junction zones between WBTs and fibers are most common, such as in *F*. *hamatacanthus*.

In fibrous species, the S/G ratio revealed that lignin consists of S and G moieties as observed in other woody dicots [29], [32], [52], [56]; however, in species with lignified rays, such as *C*. *imbricata*, *M*. *geometrizans*, and *S*. *dumortieri*, the G lignin is more abundant. It is important to note that *A*. *retusus*, a globose species with WBTs wood, has an S/G ratio similar to *L*. *marginatus* and other shrub or tree-like species with fibrous wood. It is likely that in *A*. *retusus*, highly condensed G lignin is distributed in the middle lamella between the WBT and the parenchyma and the WBT and WBT, thus the cell-cell junctions, as well as the wood, would be strong enough to resist the expansion-contraction processes. The presence of highly condensed lignin between cell-cell junctions indicates a high predominance of covalent bridges, such as those in lignin-carbohydrate complexes. These have been considered to be the compatibilizing agent between hydrophobic lignin macromolecules and hydrophilic carbohydrates, which enhance the physical and mechanical properties of wood [23].

In general, we suggest that the thick secondary walls of WBTs are richer in S lignins, which gives them sufficient flexibility to resist expansion-contraction processes caused by long periods of water stress described by other authors [1], [10], [57]. Moreover, the chemical characteristics of lignin in species with WBTs wood indicate that its main contribution is more related to the resistance to high water pressures within the cell and to the increase in flexibility rather than contributing directly to the mechanical support of the stem.

The degree of condensation in the lignin of fibrous species in Cactaceae suggests that these species have a high degradation-resistant chemical structure. However, most of dimorphic species could be more susceptible to damage caused by fungal degradation due to the high predominance of labile links between units of lignin, which has been observed in other dicots [34], [35].

## Conclusions

Our results reveal that percentage of lignin is higher in most cacti studied than any other gymnosperm and angiosperm studied. Moreover, the chemical characterization by FTIR and HPLC show that the S/G ratio in dimorphic wood is different than that found in other dicots. Moreover, the variation in the chemical composition of wood may be related to the topochemistry of the lignin as well as the abundance of fibers or WBTs. The wood anatomy in this group is very complex, and specific chemical characterization studies at the cellular level are necessary to better understand the factors involved in the large variation of the chemical composition in secondary xylem.

## Supporting Information

S1 Dataset Complete dataset of chemical composition in Cactaceae wood. Raw data and calculations.
(DOCX)

S2 Dataset Matrix for FTIR spectra. Raw data.
(DOCX)

S3 Dataset Report of S/G ratio in thirteen Cactaceae species studied by HPLC. Raw data.
(DOCX)

S1 Table Supporting information of the S/G ratio in thirteen Cactaceae species studied by HPLC. Raw data and calculations.
(DOCX)

S2 Table Supporting information of the percentage of syringyl-size plant graphic. Raw data.
(DOCX)
