# Correction: Wood Chemical Composition in Species of Cactaceae: The Relationship between Lignification and Stem Morphology

**DOI:** 10.1371/journal.pone.0130240

**Published:** 2015-06-03

**Authors:** Jorge Reyes-Rivera, Gonzalo Canché-Escamilla, Marcos Soto-Hernández, Teresa Terrazas

The Data Availability statement for this paper is incorrect. The correct statement is: All relevant data are available from Figshare. The complete dataset of chemical composition in Cactaceae wood is available under the DOI: http://dx.doi.org/10.6084/m9.figshare.1300164. The matrix for FTIR spectra is available under the DOI: http://dx.doi.org/10.6084/m9.figshare.1300167. The report of S/G ratio in thirteen Cactaceae species studie by HPLC is available under the DOI: http://dx.doi.org/10.6084/m9.figshare.1300168. Supporting Information of the S/G ratio in thirteen Cactaceae species studied by HPLC is available under the DOI: http://dx.doi.org/10.6084/m9.figshare.1300165. Supporting Information of the percentage of syringyl-size plant graphic is available under the DOI: http://dx.doi.org/10.6084/m9.figshare.1300166.
