# Supplementary material for: Wood Chemical Composition in Species of Cactaceae: The Relationship between Lignification and Stem Morphology
Source: PLoS One. 2015 Apr 16;10(4):e0123919. doi: 10.1371/journal.pone.0123919 (PMC4399841; doi:10.1371/journal.pone.0123919)
Supplement: S2 Dataset — Raw data. (DOCX) [file pone.0123919.s002.docx]

**S2 Dataset.xls** Matrix for FTIR spectra. (data)
