# Supplementary material for: Wood Chemical Composition in Species of Cactaceae: The Relationship between Lignification and Stem Morphology
Source: PLoS One. 2015 Apr 16;10(4):e0123919. doi: 10.1371/journal.pone.0123919 (PMC4399841; doi:10.1371/journal.pone.0123919)
Supplement: S1 Table — Raw data and calculations. (DOCX) [file pone.0123919.s004.docx]

**S1 Table.xls** Supporting information of the S/G ratio in thirteen Cactaceae species studied by HPLC. (data, calculations)
